# Microbial Changes during Pregnancy, Birth, and Infancy

**DOI:** 10.3389/fmicb.2016.01031

**Published:** 2016-07-14

**Authors:** Meital Nuriel-Ohayon, Hadar Neuman, Omry Koren

**Affiliations:** Faculty of Medicine, Bar-Ilan UniversitySafed, Israel

**Keywords:** microbiome, microbiota, pregnancy, infant, newborn, gut

## Abstract

Several healthy developmental processes such as pregnancy, fetal development, and infant development include a multitude of physiological changes: weight gain, hormonal, and metabolic changes, as well as immune changes. In this review, we present an additional important factor which both influences and is affected by these physiological processes—the microbiome. We summarize the known changes in microbiota composition at a variety of body sites including gut, vagina, oral cavity, and placenta, throughout pregnancy, fetal development, and early childhood. There is still a lot to be discovered; yet several pieces of research point to the healthy desired microbial changes. Future research is likely to unravel precise roles and mechanisms of the microbiota in gestation; perhaps linking the metabolic, hormonal, and immune changes together. Although some research has started to link microbial dysbiosis and specific microbial populations with unhealthy pregnancy complications, it is important to first understand the context of the natural healthy microbial changes occurring. Until recently the placenta and developing fetus were considered to be germ free, containing no apparent microbiome. We present multiple study results showing distinct microbiota compositions in the placenta and meconium, alluding to early microbial colonization. These results may change dogmas and our overall understanding of the importance and roles of microbiota from the beginning of life. We further review the main factors shaping the infant microbiome—modes of delivery, feeding, weaning, and exposure to antibiotics. Taken together, we are starting to build a broader understanding of healthy vs. abnormal microbial alterations throughout major developmental time-points.

## Introduction

Pregnancy is a remarkable biological process involving simultaneous changes in many physiological systems to support the development of healthy progeny. These changes include hormonal changes, weight gain, immune system modulation, and others, which must all be synchronized to preserve the health of both the mother and the offspring (Dunlop et al., 2015). While some of the pregnancy-associated hormonal and metabolic changes have been known for decades (Kumar and Magon, 2012), the dramatic changes in microbiome composition that take place during gestation have only recently been appreciated.

When studying the role of the microbiota in pregnancy, it is crucial to consider the stage at which the essential interaction between the host and its microbes begins. In other words, when does the developing fetus first encounter microbes? While it was hypothesized over 100 years ago that we are born germ free (Tissier, 1900) and is still the belief of many physicians, numerous pieces of evidence now cast doubt on this hypothesis, and suggest that a bacterial presence already exists in the feto-placental unit (Hu et al., 2013; Aagaard et al., 2014). The mode of birth, either vaginal or by cesarean section, was also shown to have effects on the newborn's initial microbiota, which then changes significantly as a result of the child's nutrition and overall environment throughout the first 2 years of life, until stabilization.

In this review, we describe the pronounced microbial changes that occur in the pregnant female, as well as provide an overview of the initial exposure of the fetus to microbiota—from the placenta, through birth and infancy. We hypothesize that an appropriate microbiota is essential for healthy early development, pregnancy maintenance, and the first years of childhood. Moreover, we suggest that the changes in the human microbiome are deeply connected to the host's physiological status, and that these associations have developed throughout co-evolution of microbiome and host to ensure improved fitness. Therefore, understanding the role of the microbiome throughout pregnancy and early development, as well as its role in health and disease, is of great importance for opening new research avenues, establishing developmental concepts, and perhaps even suggesting new therapeutic approaches.

## The microbiome

The human body is home to a unique microbial population, including bacteria, archaea, fungi, and viruses. The differences in microbiota composition at each body site are shaped by the varying environmental conditions such as pH, levels of oxygen, availability of nutrients, humidity, and temperature, enabling various populations to thrive and perform different functions while interacting with the human host (Ursell et al., 2012). These microbial communities have a major impact on host health, by affecting host metabolism (Turnbaugh et al., 2006), immunity (Naik et al., 2012), and hormones (Neuman et al., 2015). The majority of microbes in the human body reside in the gut, harboring hundreds of bacterial species (Lozupone et al., 2012), of which the dominant bacterial phyla are Firmicutes and Bacteroidetes (Rajilic-Stojanovic et al., 2009). Additionally, the skin, vagina, and oral cavity provide important niches for distinct bacterial communities, which contribute to the immune system by defense against potential pathogens (Naik et al., 2012).

A wide range of factors can cause shifts in the composition of the microbiota (termed dysbiosis). Dysbiosis is usually associated with harmful effects and may have long-term consequences leading to disease. Examples of disease states associated with dysbiosis include obesity, inflammatory bowel disease (IBD), diabetes, and metabolic syndrome (Spor et al., 2011). Alternatively, there are natural changes that occur in host-associated microbial composition throughout host development—from infancy to early childhood, from young to elder adults, and during pregnancy. While some of these changes may seem identical to changes seen in disease states, in their precise developmental context, they do not lead to morbidity, but rather promote fitness and survival, and may therefore be considered beneficial.

Methods to analyze the bacterial communities include culturing methods, which are limited to only a subset of species easily culturable, and more commonly used next generation sequencing techniques including 16S rRNA gene surveys and metagenomics. Data analyses from these methods can provide information on relative abundance of different microbes in a sample, as well as microbial diversity calculations both within a single sample (alpha-diversity), and between different samples (beta-diversity). Additional methods to test the microbial roles and functions include metatranscriptomics, metabolomics, and use of germ-free animal models.

Most of the data embedded in this review is focused on bacterial members of the microbiome, as they are the most researched to date. Nonetheless, it is becoming more and more evident that archaea, yeast, fungi, and viruses also affect their hosts, and we believe that future research will reveal their importance and roles in pregnancy and early life as well.

## Physiological changes during pregnancy

During gestation, the female body undergoes hormonal, immunological, and metabolic changes to support fetal growth and development (Kumar and Magon, 2012). During this period, levels of secreted hormones (especially progesterone and estrogens) rise dramatically, and there are major alterations in the immune response (Kumar and Magon, 2012). The immune changes are complex, and may be referred to as immune modulation, as on the one hand, some degree of immune suppression is needed to accept the growing fetus bearing its own developing immune system, whereas on the other hand, strict immunity must remain in order to protect the mother and fetus from infections. While many consider pregnancy to be an anti-inflammatory state, others consider pregnancy to be a multi-stage process including inflammatory stages at implantation and parturition, and anti-inflammatory stages in mid-pregnancy, when the fetus grows rapidly (Mor and Cardenas, 2010).

In many ways, the metabolic changes associated with pregnancy are similar to those that occur in the metabolic syndrome, including weight gain, elevated fasting blood-glucose levels, insulin resistance, glucose intolerance, low-grade inflammation, and changes in metabolic hormone levels (Fuglsang, 2008; Newbern and Freemark, 2011; Emanuela et al., 2012; Kumar and Magon, 2012).

**Figure 1:**
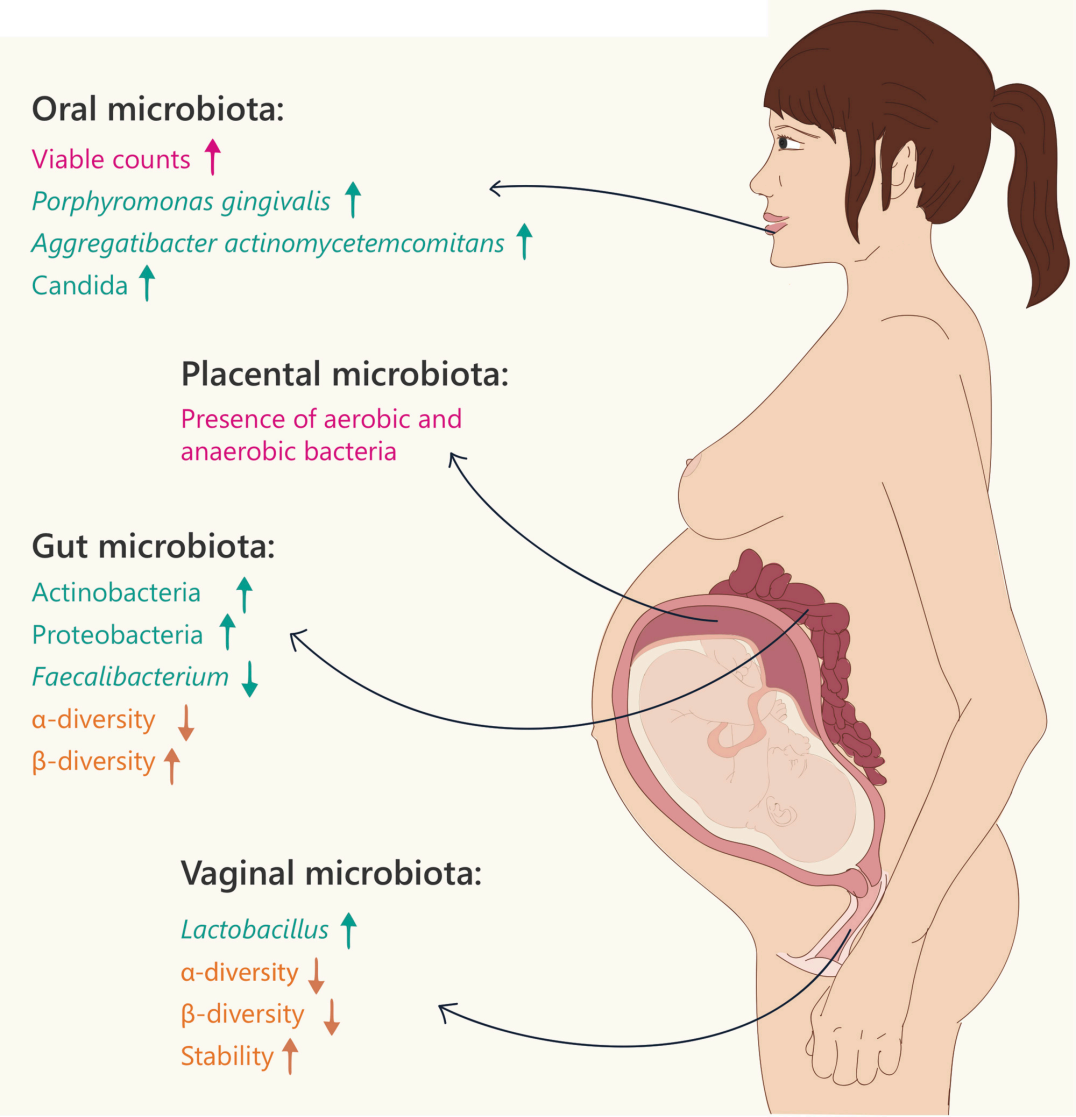
**Microbiome changes during pregnancy**. Text and arrows refer to: general changes (pink), changes in specific taxonomy (green) and community diversity (orange).

Coinciding with the endocrine, metabolic, and immune alterations, discussed above, there are noticeable changes in the microbiota at different body sites during pregnancy. These changes are discussed below, are the focus of this review and are summarized in Figure 1. However, it is important to understand the microbiota changes in the full context of all physiological changes occurring simultaneously, since regulation and cross-talk between pathways must exist.

## Changes in gut microbiota during pregnancy

Healthy pregnancy is characterized by an increase in the bacterial load and profound alterations in the composition of gut microbiota (Collado et al., 2008; Koren et al., 2012). In the first trimester of pregnancy, the gut microbial composition is similar to that of healthy, non-pregnant women. However, from the first to the third trimester, the gut microbiota composition changes dramatically. These changes are characterized by increased abundance of members of the Actinobacteria and Proteobacteria phyla, as well as a reduction in individual richness (alpha diversity; Koren et al., 2012). In addition, levels of *Faecalibacterium*, a butyrate-producing bacterium with anti-inflammatory activities, which is depleted in metabolic syndrome patients (Haro et al., 2015), are significantly decreased in the third trimester of pregnancy. Between-subject diversity (beta diversity), is increased in the third trimester, coupled with weight gain, insulin insensitivity, and higher levels of fecal cytokines, reflecting inflammation (Koren et al., 2012).

In an attempt to reveal the roles of the third trimester microbial profiles, fecal transplantation was used to transfer samples of first- and third-trimester fecal microbiotas to germ-free (GF) mice, and test their physiological effects. After transferring third trimester gut-microbiota to GF mice, the mice gained significantly more weight, developed insulin resistance, and had a greater inflammatory response compared to the first-trimester transplanted mice (Koren et al., 2012). These findings demonstrate directly that the microbial components actively contribute to changes in host immunology as well as metabolism, resembling changes seen in the metabolic syndrome. However, unlike metabolic syndrome, the bacterial composition and physical parameters in the third trimester of pregnancy have positive and essential effects in the context of pregnancy, and contribute to a healthy pregnancy, and appropriate fetal development. Just as weight gain in pregnancy is necessary to provide the needs of the growing fetus, so are the inflammatory state and insulin insensitivity. Some of the proposed mechanisms by which gut microbiota play a role in host weight gain during pregnancy include enhanced absorption of glucose and fatty acids, increased fasting-induced adipocyte factor secretion, induction of catabolic pathways, and stimulation of the immune system (Collado et al., 2008; Koren et al., 2012).

The gut microbiota during pregnancy is influenced not only by internal cues, but also by environmental factors, primarily by diet. In a recent study in mice, Gohir et al. (2015) demonstrated that the maternal diet prior to and during pregnancy had an effect on gut microbiota. Female mice fed a high-fat diet in the periconceptional period and during gestation demonstrated changes in the gut microbiota later in pregnancy, in contrast to those fed on a normal chow diet (Gohir et al., 2015). An obese state was also correlated to microbial composition during human gestation (Collado et al., 2008). *Bacteroides* and *Staphylococcus* were shown to be significantly higher in the feces of overweight pregnant women, compared to those of normal weight (Collado et al., 2008). Additionally, in overweight and obese pregnant women, specific metabolic hormones including insulin, gastric inhibitory polypeptide (GIP), and adipokines were found to be correlated with alterations in bacterial abundance, reinforcing a connection between the microbiota and metabolic hormones in pregnancy (Gomez-Arango et al., 2016). Interestingly, it was shown that pre-pregnancy maternal body mass index (BMI) correlated to neonatal gut microbiota composition in vaginally delivered, but not C-section delivered offspring (Mueller et al., 2016). Overall, most studies showed significant alterations in gut microbiota during pregnancy, with correlations to initial weight and diet, weight gain, inflammation, and metabolic parameters. An exception is a recent longitudinal study of 49 women sampled weekly, during gestation and monthly post-partum. This study found no dramatic changes during gestation or upon delivery in gut microbiota composition or richness indexes (DiGiulio et al., 2015). This strengthens the fact that more studies are needed to understand the effects of pregnancy on the gut microbiota.

The effects of antibiotics administered during pregnancy on the microbiome have been studied in rats (Khan et al., 2016). Use of category B antibiotics (azithromycin, amoxicillin, and cefaclor) during pregnancy was shown to increase the fecal relative abundance of Proteobacteria and *Enterobacter*, while reducing the relative abundance of Firmicutes and *Lactobacillus*. Moreover, antibiotics during pregnancy significantly reduced bacterial diversity, and promoted weight gain. Another study found that maternal antibiotic treatment during pregnancy and lactation in mice reduced adaptive antiviral immune responses in the infant mice, suggesting a broad immune effect on the offspring (Gonzalez-Perez et al., 2016). Most strikingly, non-absorbable antibiotics given to pregnant mice altered offspring behavior in a manner by which they exhibited low locomotor activity at 4 weeks of age, and less exploratory behavior in central regions at both 4 and 8 weeks of age. However, these behavioral phenotypes were reversed when pups were fostered by control mothers starting at postnatal day 1 (Tochitani et al., 2016).

Finally, it is intriguing to understand what are the consequences of maternal microbiome composition during pregnancy on the offspring in terms of weight gain, immunity, and infant health. A study in diet-induced obese rats revealed that oligofructose prebiotic treatment during pregnancy and lactation alters the offspring's cecal microbiome as well as prevents increased adiposity in both the dams and the offspring (Paul et al., 2016). However, it is difficult to dissect whether the main effect in this case was during pregnancy or during lactation. A study in which probiotics were administered to pregnant women 14 days before C-section revealed modulation of the infant microbiota, implying an early maternal effect on the offspring's microbiota (Rautava et al., 2012a). The same study also demonstrated a correlation between maternal probiotics and changes in expression of Toll-like receptor genes in the placenta and infant meconium. Moreover, it has been hypothesized that microbial exposure during pregnancy may be of great importance for preventing allergic disease in the offspring. The mechanisms suggested include immune-regulated epigenetic imprinting and bacterial translocation during pregnancy from the mother to the offspring, training the immune system to respond appropriately to pathogens and commensals after birth (Abrahamsson et al., 2015). Accordingly, in a study in which mothers with allergic disease and atopic sensitization received probiotics during the last 2 months of pregnancy and the first 2 months of lactation, a reduced risk for eczema in the offspring was observed compared to placebo treatment (Rautava et al., 2012b). In another study, maternal microbiota was shown to shape the offspring's immune system. Mouse pups born to GF mothers vs. GF mothers colonized with bacteria during pregnancy were compared in terms of immune gene expression and numbers of innate cells. Pups of colonized GF mothers showed increased numbers of intestinal group 3 innate lymphoid cells, F4/80^+^CD11c^+^ mononuclear cells, and increased epithelial antibacterial peptide gene expression (Rautava et al., 2012b).

The postpartum period is also characterized by significant changes in the microbiota, and it has been reported that at least 1 month after birth the mothers' microbiotas do not yet return to their baseline (Koren et al., 2012). Only a few studies have studied this timeframe, and it would therefore be of interest to investigate how long the postpartum transition period lasts, and whether a return to baseline microbial populations ever occurs. Since the postpartum period is also associated with dramatic hormonal changes including a significant decrease in progesterone and estrogen levels, it would be interesting to test the direct effects of the hormonal changes on the microbiome.

## The vaginal microbiota during pregnancy

The human vaginal microbiota is a key component in the defense system against microbial and viral infections, conferring protection against disease (Turovskiy et al., 2011). The vaginal microbiome is dominated by many species including *Lactobacillus* and members of the Clostridiales, Bacteriodales, and Actinomycetales (Aagaard et al., 2012). Within the *Lactobacillus* genus, the most frequently isolated species are: *Lactobacillus gasseri, Lactobacillus crispatus, Lactobacillus jensenii*, and *Lactobacillus iners*, which all promote various aspects of vaginal health. Specifically, these lactic acid producing bacteria can create a barrier against pathogen invasion by maintaining a low pH (< 4.5) and by secreting metabolites that play an important role in inhibition of bacterial and viral infection in the urogenital tract (Martin et al., 1999; McLean and Rosenstein, 2000). In contrast, pH-values around 5.0 were found to correlate with vaginosis (Ravel et al., 2011).

As in the gut, the vaginal microbiome undergoes significant changes during pregnancy, including a significant decrease in overall diversity, increased stability (the community composition changes over time), and enrichment with *Lactobacillus* species (Aagaard et al., 2012). These correlate with a decrease in the vaginal pH and an increase in vaginal secretions (Prince et al., 2014a). Vaginal microbial compositions were found to differ according to gestational age, while the communities at the later stages of pregnancy resembled those of the non-pregnant state (Aagaard et al., 2012). The dominant *Lactobacillus* species in pregnancy varies according to ethnic group; while *L. jensenii* is predominantly observed in women of Asian and Caucasian ethnicity, *L. gasseri* is absent in samples from Black women (MacIntyre et al., 2015).

A study characterizing the vaginal microbiota of pregnant and non-pregnant African-Americans reported that during pregnancy, one of the changes is dominance of a single *Lactobacillus* species over others (Romero et al., 2014a). This may be due to the fact that some *Lactobacillus* species have bactericidal activities against other species, ensuring their predominance and low variability, which may help in protection against infections during pregnancy (Spurbeck and Arvidson, 2010).

Contrastingly, a recent study by DiGiulio et al. (2015) did not identify any significant alteration in vaginal microbial components throughout pregnancy. However, they found distinct differences in the post-partum period in which vaginal communities were more similar to the gut microbiota communities, with *Lactobacillus* being replaced by various anaerobic bacteria, including *Peptoniphilus, Prevotella*, and *Anaerococcus*. These various alterations may persist for as long as a year after delivery (DiGiulio et al., 2015). The postpartum vaginal microbiome was also characterized by gradual depletion of *Lactobacillus* species, increased alpha-diversity, and enrichment of bacteria associated with vaginosis, such as *Actinobacteria*, in 40% of the subjects at 6 weeks after birth (as opposed to only 2% of the subjects during pregnancy; MacIntyre et al., 2015).

## The oral microbiota during pregnancy

The oral microbiome includes up to 600 diverse species including *Streptococci, Lactobacilli, Staphylococci, Corynebacteria*, etc., residing in different microenvironments within the oral cavity (teeth, tongue, palates, etc., Dewhirst et al., 2010). When comparing the abundance of seven common bacterial species in the oral cavity of non-pregnant women, early pregnancy, mid-pregnancy, and late pregnancy, the total viable microbial counts in all stages of pregnancy were higher than those of the non-pregnant women, especially in early pregnancy (Fujiwara et al., 2015), and levels of the pathogenic bacteria *Porphyromonas gingivalis* and *Aggregatibacter actinomycetemcomitans* in the subgingival plaque, were significantly higher during the early and middle stages of pregnancy, compared to the non-pregnant group (Fujiwara et al., 2015). These results were further reinforced in an additional study, showing higher levels of *A. actinomycetemcomitans* in the second and third trimesters of pregnancy compared to non-pregnant women (Borgo et al., 2014). Additionally, *Candida* levels were significantly higher during middle and late pregnancy compared to non-pregnant women, further demonstrating a higher prevalence of periodontal pathogens in pregnancy.

While some efforts have been made to elucidate mechanisms by which pregnancy leads to changes in the oral composition, these pathways remain unclear. It has been suggested that progesterone and estrogen affect the microbiota during pregnancy, but these effects have not been fully deciphered nor directly proven, other than the finding that estrogens enhance *Candida* infections (Kumar, 2013; Fujiwara et al., 2015). It is likely that the overall immune state during pregnancy plays a role leading to increased oral microbial load. Intriguingly, numerous correlations have been suggested between oral disease and pregnancy complications and outcomes, as described below.

## The placental microbiota

The placenta is one of the most poorly understood human organs, particularly with regard to the presence of microbes within it. Until recently, the dogma was that the fetus and the placenta are germ free, and that such a sterile environment is essential to protect the fetus from infections. It was believed that cases of placental contamination were caused by infection originating from the lower genital tract (Goldenberg et al., 2000). However, several lines of evidence suggest the presence of bacteria in the normal placenta, itself.

In 1982, Kovalovszki et al. were the first to describe the presence of aerobic bacteria in 16% of tested placenta samples using a culture-dependent approach, and concluded that bacteria can be detected in the placenta without any histological evidence of chorioamnionitis (Kovalovszki et al., 1982). Since then, several additional reports described bacteria in the placenta (Goldenberg et al., 2000; Dominguez-Bello et al., 2010; Hyman et al., 2014; Romero et al., 2014a; MacIntyre et al., 2015). Using whole genome shotgun sequencing (WGS) of samples from 320 subjects, Aagaard et al. reported that the placenta contains a unique microbiome (Aagaard et al., 2014). When characterizing the placental microbial community, the major phylum was Proteobacteria, and compared to all other organs, the composition was most similar to the oral microbiota, including species such as *Prevotella tannerae* and *Neisseria* (Aagaard et al., 2014). The similarity between the oral and placental microbiota suggests that bacteria may pass from the oral cavity to the placenta, possibly explaining the many observations of women with periodontal disease that have an increased risk of pregnancy complications (Gibbs et al., 1992; Jefferson, 2012; Mysorekar and Cao, 2014; Prince et al., 2014b). Additionally, several studies (Figure 1), using cultivation and PCR techniques, found microbes in amniotic fluid and umbilical cord blood in healthy asymptomatic women, as well as in those with pregnancy complications (DiGiulio et al., 2010a,b; Romero et al., 2014b). It is important to note that the microbial density present in the placenta is very low, requiring precise verification in all cases that research results are not due to contamination. This is especially true with samples in which the placenta was collected after vaginal delivery and is therefore prone to contaminations from the birth canal, as opposed to samples in which the placenta was collected during C-section in a sterile manner.

Nonetheless, these initial findings posit further questions regarding the localization of the bacteria within the placenta, and whether different bacterial populations are present at different placental sites, as the placenta includes different niches, both in terms of interaction with the maternal vs. fetal circulation, and oxygen levels. Another intriguing issue is whether microbial components are necessary for healthy placental development and function, and the underlying mechanisms of these effects.

## The role of dysbiosis and infections in pregnancy complications

Pregnancy complications such as preeclampsia, eclampsia, intrauterine fetal death, intra-uterine growth restriction (IUGR), placental abruption, and preterm birth, are observed in approximately one in every six pregnancies, and can impact both maternal and fetal health and survival (Kujovich, 2004). Although these complications affect many pregnant women worldwide, the etiology in most cases is unknown (Rizzo and Arduini, 2009). It has long been suggested that bacterial infections may be correlated with pregnancy complications, and may even induce these states (Seong et al., 2008). With the emergence of microbiome research and the realization that changes in the maternal microbiome compositions occur during pregnancy, it has been suggested that there may be correlations between the microbial communities present, and the health of the pregnancy (Mysorekar and Cao, 2014). Thus, the search began for microbial markers associated with pregnancy complications. In this regard, it is important to distinguish between the normal and healthy microbial changes that occur during pregnancy, vs. undesired changes, which may be linked to pregnancy complication states.

When studying the vaginal microbiome, Romero et al. did not find any association between the degree of alpha diversity and pregnancy outcomes (Romero et al., 2014a), while two other studies did demonstrate a correlation between high alpha diversity and preterm birth (Hyman et al., 2014). DiGiulio and colleagues identified candidate vaginal communities in the early stages of pregnancy that were significantly associated with an elevated rate of preterm birth (DiGiulio et al., 2015). These communities include higher abundances of *Gardnerella* and *Ureaplasma*, lower abundances of *Lactobacillus* sp., and higher alpha diversity. Mechanistically, abnormal changes in the vaginal microbiota during pregnancy, such as decreased levels of *Lactobacillus* sp., may lead to infections, and production of pro-inflammatory cytokines, and prostaglandins, which can cause uterine contractions and weaken fetal membranes (Park et al., 2005; Lajos et al., 2008). Additionally, presence of certain fungi, such as vaginal colonization of *Candida albicans* even when asymptomic, is correlated to higher rates of preterm birth (Farr et al., 2015). Although the effects are not direct, it was suggested that beyond the healthy changes related to pregnancy, gut dysbiosis may induce pregnancy complications by impairing maternal adaptation (Zhang et al., 2015).

Correlations between oral infections and pregnancy complications have been shown in various studies (Zi et al., 2014). Maternal periodontal disease has been shown to increase risk of preterm birth (Offenbacher et al., 2006). While at first, the connection between oral infections and pregnancy complications may sound farfetched; there are several mechanisms by which such a link may occur. One theory posits that lipopolysaccharides (LPS) from Gram-negative periodontopathic bacteria such as *Porphyromonas gingivalis* may increase inflammatory mediators and prostaglandin production, leading to preterm birth (ElAttar, 1976; Kim and Amar, 2006). Alternatively, it was suggested that hematogenous spread of oral microbes to the placenta might be a driver of preterm birth (Aagaard et al., 2014).

Finally, as evidence for a placental microbiome is accumulating, one emerging question is whether these bacterial communities are different in cases of pregnancy complications such as preterm births. When comparing placental bacterial populations in preterm vs. term deliveries, higher abundance of several taxa, such as *Burkholderia, Streptosporangium*, and *Anaeromyxobacter*, were found in the placentas of pregnancies delivered preterm, while *Paenibacillus* was enriched in placentas delivered at term (Romero et al., 2014a). However, using histologic stains, no significant differences were seen between preterm and term placentas (Stout et al., 2013). While some of these studies indicate changes in bacterial composition of the placenta correlating to preterm deliveries, more research is needed to validate microbial placental signatures associated with preterm birth.

## Early bacterial colonization of the fetus

Until recently, it was assumed that newborns are born completely germ free, and that the initial colonization of the newborn gut occurs during birth. However, several studies suggest that the colonization of the fetus begins prior to birth. In 2008, Jimenez et al. orally inoculated pregnant mice with a genetically labeled *Enterococcus faecium* strain and, afterward, were able to isolate this bacterium from the meconium (first stool) of offspring delivered by C-section (no labeled bacteria were found in the uninoculated control group; Jimenez et al., 2008). This finding implies that maternal bacteria can enter the GI tract of the fetus. However, direct proof for microbial colonization of the fetus, as well as the potential mechanisms by which bacteria pass from the mother to the fetus are still unknown. In a human study in which pregnant mothers received probiotics compared to placebo controls, bacterial alterations were detected in the infant meconium, and placenta (Rautava et al., 2012b) once again alluding to a prenatal effect on microbial composition. The notion of early microbial colonization of the fetus suggests that from the very beginning of development, there are dual interactions between the developing host and microbiota, and that there is some type of early inheritance of the maternal microbial components.

## The meconium microbiome

Just like the placenta and fetus, the meconium was considered in the past to be sterile (Koleva et al., 2015), however several recent studies have shown using a variety of methods presence of bacterial populations including *Enterococcus, Escherichia, Leuconostoc, Lactococcus*, and *Streptococcus* (Dominguez-Bello et al., 2010; Gosalbes et al., 2013; Hansen et al., 2015) Compared to adult fecal samples, the infant meconium shows lower species diversity, higher between-subject variation, and enrichment of Proteobacteria at the expense of reduction of Bacteroidetes (Hu et al., 2013). Ardissone et al. (2014) found a correlation between newborn gestational age and meconium microbial compositions. Specifically, the *Enterobacter, Enterococcus, Lactobacillus, Photorhabdus*, and *Tannerela* genera were more abundant in the preterm infants' meconium (Ardissone et al., 2014).

## Mode of delivery and the newborn microbiome

In 2013, Gosalbes et al. found no significant differences between the meconium microbiota of vaginal vs. Cesarean section (C-section) delivered infants (Gosalbes et al., 2013). However, several additional studies have shown distinct effects of delivery mode on the gut microbiota composition of newborn babies. Generally, the gut microbiota of vaginally born infants is first colonized by bacteria from the maternal vagina, characterized by enrichment in the *Prevotella, Sneathia*, and *Lactobacillus* genera, and also includes bacteria present in the maternal gut (Mackie et al., 1999; Dominguez-Bello et al., 2010). In contrast, the gut microbiota of infants born by C-section indicates a relative resemblance to maternal skin and oral microbiota dominated by *Propionibacterium, Corynebacterium*, and *Streptococcus* (Backhed et al., 2015; MacIntyre et al., 2015). The infants born by C-section also exhibit delayed colonization of the phylum Bacteroidetes, and lower alpha diversity during the first 2 years of life (Jakobsson et al., 2014). However, the differences in species diversity between delivery modes are decreased after 4 months, and almost disappear by 12 months of age (Backhed et al., 2015). The microbiome of C-section delivered infants was also found to contain a higher proportion of bacterial antibiotic-resistance genes compared to vaginally delivered infants. This, together with a higher abundance of *Staphylococcus* in C-section newborns compared to vaginal births, may explain why 64–82% of reported cases of methicillin-resistant *Staphylococcus aureus* (MRSA) skin infections in newborns occur in C-section delivered infants (Centers for Disease Prevention, 2006).

Interestingly, the mode of delivery has an effect on the infant's oral microbiome, as well (Lif Holgerson et al., 2011). Immediately after delivery, the oral microbial communities of C-section and vaginally delivered newborns resemble that of the mother's skin or vagina, respectively (Dominguez-Bello et al., 2010). It was further shown that the oral bacterial populations differ at 3 months of age between C-section and vaginally delivered infants. Generally, more bacterial taxa were detected in the infants delivered vaginally compared with infants delivered by C-section, with *Slackia exigua* detected only in infants delivered by C-section. In a different study of infants infected with *S. mutans*, involved in dental caries, those delivered by C-section acquired infection almost 1 year earlier than did vaginally delivered infants, and the genotype of these bacteria, was similar to that of their mothers (Li et al., 2005).

It has been suggested that disruption of vaginal bacteria transmission via C-section can have long-term medical implications: a few studies reported that C-section delivery increases the incidence of celiac disease (Decker et al., 2010; Marild et al., 2012), obesity and asthma (Kero et al., 2002). Moreover, the administration of probiotics (including *Lactobacillus* sp.) from birth until age 6 months reduced the incidence of allergy at 5 years of age in C-section, but not vaginally delivered children (Kuitunen et al., 2009). However, none of these studies directly tested the role of bacteria colonizing the newborn in the development of disease. The mode of delivery may also have an effect on the maturation of the immune system. Jakobsson et al. found lower levels of the T helper cell-related chemokines CXCL10 and CXCL11 in the blood of infants delivered by C-section compared with vaginally born infants. Moreover, bacteria from the genus *Bacteroides* (which are more abundant after vaginal delivery) affect the maturity of the immune system (Kelly et al., 2004), suggesting that C-section may lead to immune disorders.

The distinct differences between the microbiome compositions of C-section vs. vaginal delivered infants have led scientists to expose C-section born infants to vaginal fluids containing microbiota at birth and test the effects on the infant's microbial composition up to 30 days later (Dominguez-Bello et al., 2016). When comparing the gut, oral, and skin bacterial communities of the C-section delivered babies, those exposed to vaginal fluids revealed significantly more vagina-associated bacteria, making their compositions more similar to vaginally born infants.

**Figure 2:**
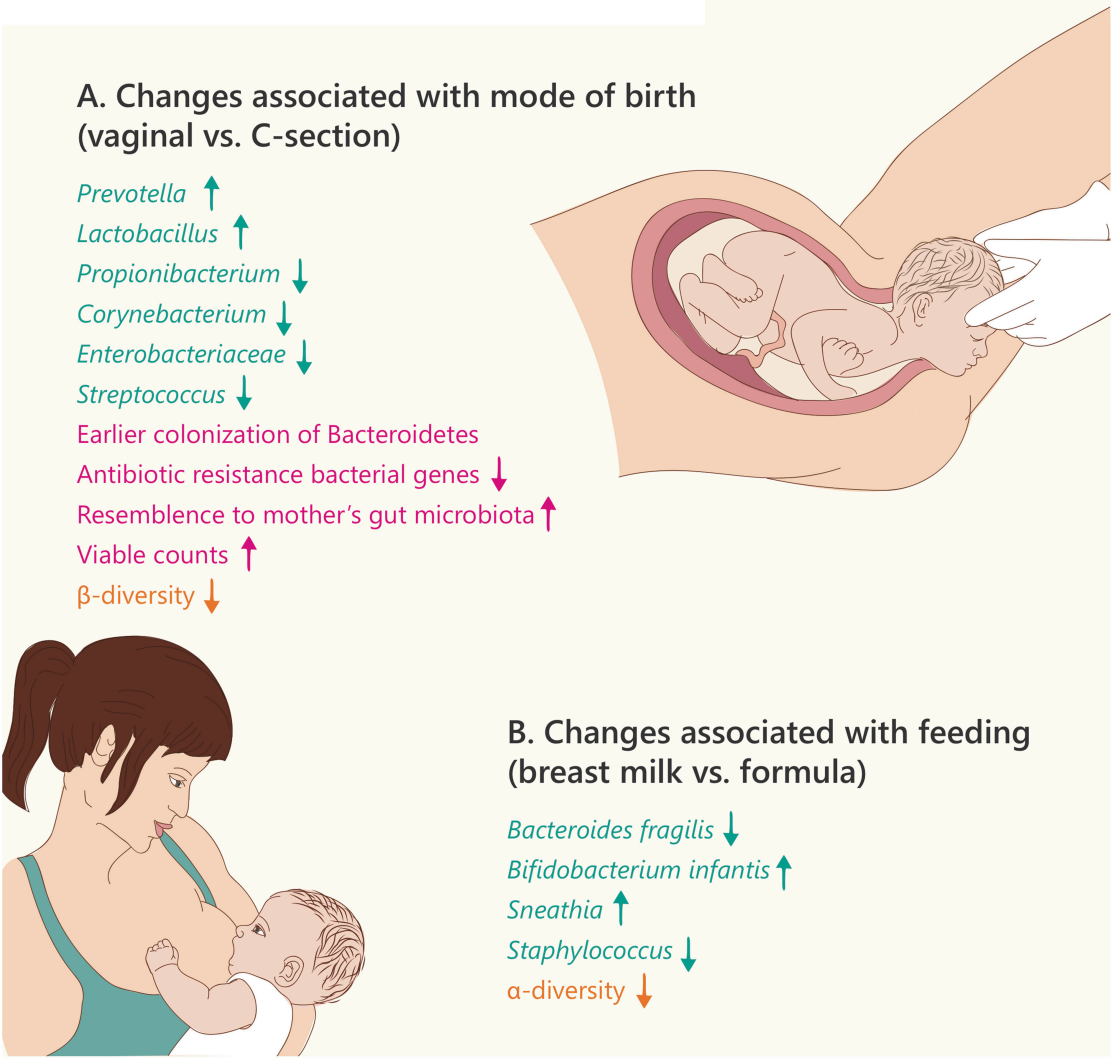
**Microbiome changes in the newborn**. Text and arrows refer to: changes in specific taxonomy (green), general changes (pink), and community diversity (orange). **(A)** Text refers to changes in vaginal birth vs. Caesarian section **(B)**. Text refers to changes in breast milk vs. formula feeding.

In summary, it appears evident that the mode of birth affects the newborn's microbiome composition (Figure 2). It still remains unclear if these different bacterial communities in infancy have long-term effects in terms of microbiome composition, metabolism, and overall health.

## Gut microbiota during the first 2 years of life

From the time of birth, infants acquire microbes from their environment, from their diet, and from people surrounding them. The first 2 years of life, which are characterized by enormous dietary changes, new environmental exposures, and maturation of the immune system therefore have a strong influence on the infant's microbiota. The two most important factors found to shape the infant gut microbiota are mode of birth and feeding (breastmilk vs. formula and then solid foods; Figure 2). In the first months of life, the gut microbiota is less stable than later in childhood, and there are changes in the phylogenetic diversity. Koenig et al. showed, by following an infant over a 2.5-year period, that the alpha diversity of the infant's gut microbiota gradually increases over time (Koenig et al., 2011). However, the high β-diversity observed at birth (which is higher than that observed in adults) is reduced by 12 months of age (Backhed et al., 2015). In terms of function, at 12 months of age there is more similarity to the mother's gut metagenome, compared to newborns, implying that the microbiome matures during the first year of life. Specifically, as the nutrition and energy sources change throughout the first year, so do the microbial energy utilization components: while microbial genes required for degradation of sugars from milk are most abundant at 4 months, microbial genes degrading complex sugars and starch are more abundant at 12 months (Backhed et al., 2015).

Additionally, illness and environmental changes such as fever, antibiotic treatment, and changes in diet can cause large shifts in the bacterial compositions and abundance of some taxonomic groups (Koenig et al., 2011). The differences in microbiota throughout development of the healthy infant can be plotted to show the gradual changes according to age. Malnutrition in young children was shown to affect their microbiome causing it to resemble that of earlier developmental stages (Smith et al., 2013).

## The human milk microbiome

Human milk is produced by the mother to promote the normal and healthy development of her infant offspring. This fluid is considered to be the optimal source for all the nutrition factors that the infant needs, containing many proteins, lipids, and 200 types of oligosaccharides (carbohydrates) that are not found elsewhere (Kunz et al., 2000). Until recently, the prevailing paradigm was that milk was sterile; however, recent studies have proven otherwise by showing that human milk not only contains bacteria, but is the predominant source for establishing a “healthy microbiome” in the newborn (Heikkila and Saris, 2003; Martin et al., 2007, 2009). In 2011, Hunt et al. found that the milk microbiome is extremely diverse, and suggested a “core human milk microbiome” after finding nine operational taxonomic units (OTUs) consistently over samples from 16 subjects (Hunt et al., 2011). Further characterization revealed that the microbial composition changes over the course of the lactation period. The most dominant bacteria in the colostrum (the first milk produced during late pregnancy, and contains antibodies) included *Weisella, Leuconostoc, Staphylococcus, Streptococcus*, and *Lactococcus*, whereas milk samples collected 1–6 months post-partum harbored oral cavity related bacteria such as *Veillonella, Leptotrichia*, and *Prevotella*, perhaps due to frequent interaction with the infant's oral microbiota (Cabrera-Rubio et al., 2012).

Maternal weight was found to be a major influencing factor in shaping milk bacterial composition. In obese pregnant women, higher abundance of the genus *Staphylococcus*, namely *S. aureus* was reported over the first 6 months of lactation, as well as higher abundance of *Lactobacillus* in the first month, compared with samples from mothers of normal weight (Cabrera-Rubio et al., 2012). Interestingly, high levels of *S. aureus* are also found in the gut microbiota of overweight children; however, the source of this bacterium is, as yet, unclear (Kalliomaki et al., 2008). Future research is needed to determine whether the milk microbial populations associated with maternal obesity may have implications on the offspring's weight.

The source of the milk microbial populations is considered to be from the gut, and the mechanism by which bacteria travel from the gut into the mammary glands is thought to be facilitated by hormonal changes that occur during and following gestation. More specifically, elevation of progesterone may increase gut permeability, which could assist in bacterial transmission into the bloodstream and subsequently into the mother's mammary glands (Jeurink et al., 2013). It will be interesting to further test the function and importance of the milk microbiome—whether these bacteria play roles in milk metabolism or immunity, and to what extent they affect the infant's microbial populations.

## Effects of breast-feeding vs. formula on infants

There are profound differences in the gut microbiota composition of breast-fed vs. formula-fed infants (summarized in Figure 2B). Breast milk contains oligosaccharides, complex carbohydrates that cannot be digested by the infant. However, some of the infant's bacteria including *Bifidobacterium, Lactobacillus*, and *Bacteroides* have the ability to degrade these oligosaccharides into small sugars that may be utilized as an energy source (e.g., lactose), giving them an advantage over competing bacteria (Sela et al., 2011; Manthey et al., 2014). The remaining small sugars can be further metabolized by glycolytic microbes present in the infant, including lactate-producing bacteria such as *Streptococcus, Staphylococcus*, and *Enterococcus*. This amazing co-evolution of the maternal milk biosynthesis and the infant's microbiota components that can beneficially utilize it, enhances the nutrition of the young holobiont (Bode, 2012).

Studies in the last two decades revealed that *Bifidobacteria* are the most abundant organisms in breast-fed infant guts, whereas the gut microbiota of formula-fed infants is dominated by *Enterococci* and *Clostridia* (Balmer and Wharton, 1989). Furthermore, Bezirtzoglou et al. observed that the gut of formula-fed infant contained fewer bacterial cells and more species diversity compared to that of breast-fed infants (Bezirtzoglou et al., 2011).

Not surprisingly, the method of feeding (breast-feeding or infant formula) affects the infant's oral microbiome as well. Three-month old breast-fed infants contained oral lactobacilli with antimicrobial properties that were not found in formula-fed infants (Holgerson et al., 2013; Vestman et al., 2013).

The weaning period with transition to solid foods has a significant role in shaping the gut bacterial composition. After weaning, the infant's gut microbiota is enriched with species that are prevalent in adults, including Bacteroidetes and Firmicutes from the Clostridia class: *Clostridium, Ruminococcus, Faecalibacterium, Roseburia*, and *Anaerostipes* (Valles et al., 2014; Backhed et al., 2015). Additionally, levels of fecal short chain fatty acids were shown to be higher after introducing solid foods to infants (Koenig et al., 2011). The shift to solid food, as well as increased exposure to xenobiotics, change the microbial populations and enriches species which are able to utilize nutrients such as carbohydrates, and are involved in vitamin biosynthesis and xenobiotic degradation, thereby maturing the gut microbiome (Backhed et al., 2015).

In summary, there appear to be significant differences in microbiota composition depending on diet and feeding mode. However, further research is needed to fully understand the meaning of these distinctions, and to test whether they have any long-term effects.

## Conclusions

In this review, we discuss the microbiome compositions during three crucial developmental stages: pregnancy, birth, and infancy. These stages are accompanied by numerous physiological changes, including changes in hormonal levels, inflammation, and metabolic states. Pregnancy and infancy are both times of growth and increased weight gain, changes in diet, and alterations in immune system components. The birth process itself includes many simultaneous environmental and developmental changes, among them detachment from the placenta, initiation of breathing, exposure to air and outer environment, and active use of the GI tract. Obviously, these numerous events and changes must be synchronized in order to achieve the healthy normal development enabling life.

We describe significant alterations in microbial compositions in a variety of body sites (gut, vagina, placenta, breast milk, etc.) throughout these developmental processes. However, much work remains to decipher the meaning of these changes, and their role within the overall process leading to healthy pregnancy, birth, and early development. We believe that there is significance to the microbial changes seen throughout development, and that additional research will allow this puzzle to be solved. However, it may be impossible to decipher the meaning of the changes without the full developmental context. For example, similar characteristics and changes occur in pregnancy as in metabolic syndrome, including dysbiosis, inflammation, and weight gain. In both cases, the bacteria affect host metabolism and immunity; however the developmental context is crucial for differentiating between the “beneficial” nature of these changes in pregnancy, as they promote energy storage in fat tissue and growth of the fetus, as opposed to their “harmful” nature in the context of metabolic syndrome.

The complex interactions between the host and the microbiota during pregnancy and early development remains to be fully understood. Most of the studies presented in this review are association studies, highlighting changes in microbiota composition, richness, and diversity between groups. However, few studies analyzed the actual effects of microbial changes on host development (e.g., by fecal transplant experiments), or even the metabolomics and transcriptional affects. It seems likely that there is no simple answer to the question of whether the microbiome affects or is affected by pregnancy, birth, and early development, and it appears that the effects are bidirectional.

When thinking of pregnancy, one of the first and perhaps most important changes that occur are hormonal changes. In this regard, it has already been shown that the microbiome both affects and is affected by the endocrine system. The microbiota has the ability to produce and secrete hormones or hormone analogs, and respond to and regulate host hormones. On the other hand, host hormone levels can influence bacterial growth, rendering the interplay between microbiota and hormones bidirectional. Nonetheless, precise data on the effects of female hormones on the microbiota and vice versa are still needed.

Another complex interconnection is found between the microbiome and immune system. During pregnancy, the immune system must be modulated to allow for proper fetal development (including development of the fetal immune system), as well as preventing fetal rejection by the maternal immune system. The microbial components are greatly affected by immune modulation, but are also active players in the system.

Finally, there are dual effects between the microbiome and host metabolism. The microbiota is both affected by the host's metabolic state (including dietary components and host hormones), as well as affecting host metabolism (in terms of energy homeostasis, fat storage, and hormonal regulation). This can be demonstrated by fecal transplantation to GF mice, leading to phenotypes resembling metabolic syndrome (Harley and Karp, 2012; Koren et al., 2012).

Identifying the beneficial and detrimental microbial components and their roles during pregnancy, parturition, post-partum, and in early development may have extremely important implications in terms of diet, antibiotic treatments, probiotics, and potential therapies for disease states throughout all developmental stages. Therefore, the microbial interactions during these times may have clinical relevance in the design of the desired type of antibiotics and/or probiotics for pregnant women or newborns. For instance, if specific microbial species in human milk prove to have long-term health benefits, they may be administered to formula-fed newborns, as well. Alternatively, probiotic or antibiotic treatments could be designed to alleviate pregnancy complication states, or at least, microbial biomarkers could be identified for the early diagnosis of such conditions. We are therefore confident that future research in these fields will provide useful clinical knowledge, as well as a broader understanding of healthy vs. abnormal development.

## Author contributions

MO, HN, and OK reviewed the literature and wrote the manuscript.

## Funding

The work by MO and OK was supported by the Marie Curie International Reintegration Grant (FP7-PEOPLE-2013-CIG-630956) and the Ministry of Health, State of Israel (3-0000-10451). HN was supported by Ministry of Science, Technology & Space, State of Israel. (3-11714)

### Conflict of interest statement

The authors declare that the research was conducted in the absence of any commercial or financial relationships that could be construed as a potential conflict of interest.
